# Electrically tunable solid-state metasurfaces realized by flash localized heating

**DOI:** 10.1038/s41377-023-01165-8

**Published:** 2023-05-17

**Authors:** Qian Sun, Minghui Hong

**Affiliations:** Pen-Tung Sah Institute of Micro-Nano Science and Technology, Xiamen University, Xiamen, 361005 China

**Keywords:** Optics and photonics, Metamaterials

## Abstract

Electrically programmed metasurfaces provide large modulation depth, high modulation rate, and solid-state component, breaking the limitations of existing modulation methods.

Traditional optical components modulate the wavefront by using the transmission phase accumulation generated by light propagation in the medium. The modulation capability is limited by the modulation mechanism and material properties. The volume is relatively bulky and the function is single, which is difficult to satisfy the miniaturization and integration of optoelectronic devices. Metasurface^1^ holds promise for solving this problem based on the generalized refraction law, which is presented as a two-dimensional ultra-thin array plane with artificial subwavelength structures. It can realize complex optical modulation functions by pixelated phase or amplitude modulation on the nanometer scale, which promotes the integration of optical devices in principle.

In recent years, metasurfaces have been widely studied and developed. Typically, the metasurface is designed and manufactured with a fixed optical property, which cannot satisfy the demand of complicated applications, such as being used in beam scanning as zoom lens. Therefore, researchers proposed a novel metasurface with active tuning capability to enhance its practicability. The optical properties of the metasurfaces are expected to be controlled dynamically by introducing external field modulation. At present, potential approaches for dynamic modulation have been reported, such as phase-change materials^2^, liquid crystals^3,4^, semiconductor materials^5,6^, two-dimensional materials^7–9^, and micro-electromechanical system-based actuators^10^. However, the existing modulation methods still face many challenges, such as low tuning rate, weak modulation, and non-solid-state components. Therefore, a new tuning method is required to solve these problems.

In this issue of Light: Science & Applications, Dragomir Neshev, Mohsen Rahmani, and their colleagues^11^ demonstrated a novel electrically tunable metasurface, breaking through the limitations of existing modulation methods.

In this work, the authors introduced an effective method of adapting fast electrical switching by an optical switch, which uses thermo-optical silicon membrane metasurfaces, controlled by electrically driven localized transparent heaters. The schematic of the device is shown in Fig. 1, it consists of a transparent conductive oxide-encapsulated silicon hole array metasurface. When an electric potential difference is applied to the contact, electrons start to travel in the conductive indium tin oxide strip, which creates local heaters on the desired pixel metasurfaces. The temperature of the heater can be controlled by an electronic switch in the drive system. Due to the thermo-optical effect^12^, an increase in the metasurface temperature leads to a change in the refractive index of the resonators, which in turn leads to a shift in the resonant wavelength. Therefore, this research converts the electrical switch to local flash heating, which consequently converts to an optical switch.

**Fig. 1:**
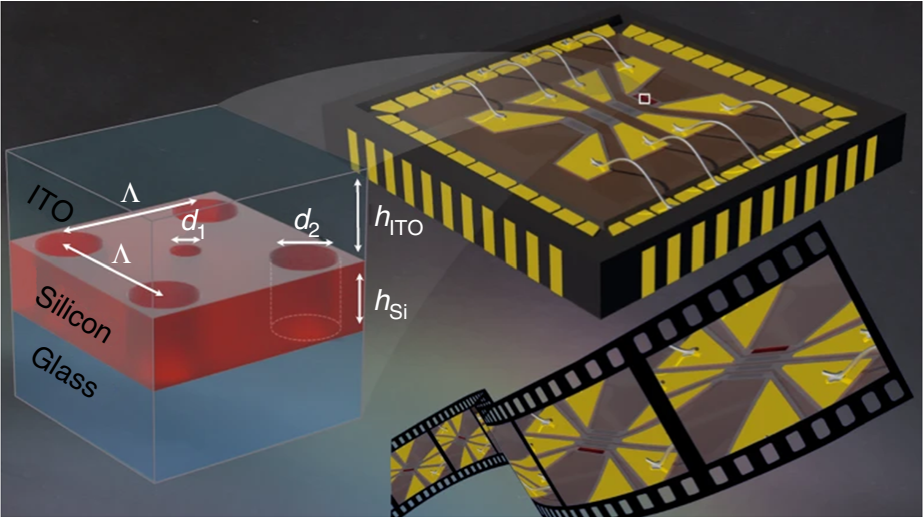
Electrically switchable metasurface pixels by flash localized heating. Illustration of the fabricated sample, composed of designed nanoholes in a silicon film, so-called hole array metasurfaces, embedded in an ITO transparent heater. The parameters of the design are h_Si_ = 155 nm, d_1_ = 78 nm, d_2_ = 101 nm, h_ITO_ = 380 nm, and pitch Λ = 350 nm

The designed electrically tunable metasurface exhibits impressive characteristics. This work creatively adopts an asymmetric voltage spike to achieve up to 90% optical modulation depth with the switching time <625 μs. It indicates that the device can work at a switching rate much higher than the video frame rate, demonstrating the ultra-high modulation rate and large modulation depth of the metasurface. Meanwhile, pure solid-state components are employed, which are more robust and stable compared to liquid crystal tunable metasurfaces. Furthermore, the designed metasurface is electronically programmable and CMOS-compatible, improving the efficiency and applicability of the metasurface.

This innovative technology for electrically tunable metasurfaces is strategically important for the development of optical switches. The proposed mechanism fills up the technique blanks for the tunable metasurfaces capable of switching light effectively in the transmission regime at high frequencies. The development of the metasurface is thus a critical step forward, which will provide unprecedented light manipulation capabilities. This research will have promising applications in flat displays, lidar systems, optoelectronic detection systems, augmented reality devices, and other fields.
